# Understanding the Burdens Associated with Huntington’s Disease in Manifest Patients and Care Partners–Comparing to Parkinson’s Disease and the General Population

**DOI:** 10.3390/brainsci12020161

**Published:** 2022-01-26

**Authors:** Alex Exuzides, Joana E. Matos, Anisha M. Patel, Ashley A. Martin, Bryan Ricker, Danny Bega

**Affiliations:** 1 Genentech Inc., South San Francisco, CA 94080, USA; exuzides.alex@gene.com; 2 Cerner Enviza, North Kansas City, MO 64117, USA; matos.joanae@gmail.com (J.E.M.); ashley.martin@cernerenviza.com (A.A.M.); Bryan.Ricker@cernerenviza.com (B.R.); 3 Department of Neurology, Feinberg School of Medicine, Northwestern University, Chicago, IL 60611, USA; danny.bega@nm.org

**Keywords:** Huntington’s disease, Parkinson’s disease, anxiety, depression, caregiver

## Abstract

Background: The study provides real-world data on the impact of Huntington’s disease (HD) from the perspective of individuals with HD (IHD) and care partners (HD-CP) and contextualizes these results relative to Parkinson’s disease (PD) and the general population (GP). Methods: Cross-sectional survey of IHD and HD-CP in the US (July 2019–August 2019) conducted using the Rare Patient Voice panel. Data for individuals with Parkinson’s Disease (IPD), the general population (GP), and respective care partners (PD-CP; GP-CP) came from the 2018 US National Health and Wellness Survey. Outcomes included demographics, mental health, clinical characteristics, and health-related quality of life (HRQoL). Results: IHD had greater comorbid anxiety (IHD = 51.2%, IPD = 28.8%, GP = 2.0%), and HD-CP had greater comorbid anxiety (HD-CP = 52.5%, PD-CP = 28.6%, GP-CP = 19.6%) and depression (HD-CP = 65.0%, PD-CP = 29.9%, GP-CP = 19.6%), relative to other cohorts (*p* < 0.05). Respective of their GP cohorts, IHD exhibited lower HRQoL (EQ-5D: 0.66 ± 0.21 vs. 0.81 ± 0.17) and greater depression (PHQ-9: 11.59 ± 7.20 vs. 5.85 ± 6.71), whereas HD-CP exhibited greater depression only (PHQ-9: 6.84 ± 6.38 vs. 4.15 ± 5.58) (*p* < 0.001). No differences were observed between HD/HD-CP and PD/PD-CP cohorts on PHQ-9 or HRQoL. Conclusions: HD has a significant burden on patients and care partners, which is higher than GP. Notably, anxiety and depression were greater among HD vs. PD, despite similar HRQoL.

## 1. Introduction

Huntington’s disease (HD) is a progressive, autosomal dominant, inherited neurodegenerative disorder affecting as many as 13.7 per 100,000 individuals based on worldwide prevalence estimates [1,2,3]. The disease is caused by an expanded CAG triplet repeat in the huntingtin gene (HTT), which in turn leads to expression of a mutated toxic huntingtin protein [4]. Accumulation of this mutated protein leads to the gradual development of motor abnormalities (e.g., chorea, dystonia, dysarthria), progressive deterioration of cognitive processes (e.g., executive dysfunction, emotional regulation), and a variety of neuropsychiatric symptoms such as depression, which is considered a central component of HD [5] and affects an estimated 50% of patients [1,6]. Typically, the onset of HD occurs in mid-life between 30–50 years of age, though symptoms can manifest as early as age 5 and as late as age 80+ [7].

Due to the progressive nature of the disease, symptoms worsen over the lifespan, leading to increased dependency on care partners for performing daily activities [1,8]. Thus, HD confers increasing burden over time, both to patients (as a result of their worsening symptomatology) as well as to those who care for them. These humanistic burdens are myriad, impacting mental health, financial wellbeing, and inter-personal relationships [9,10,11,12,13,14,15].

While the impacts of HD on patients and care partners have been a focus of prior research, these studies mostly describe the burdens in isolation, without contextualizing the experience of HD patients and care partners relative to those without neurodegenerative disease, or to those with a differing neurodegenerative disease. For example, Parkinson’s disease (PD) is another progressive neurodegenerative condition that affects as many as 418 per 100,000 individuals based on worldwide prevalence estimates [16]. The disease is caused by the death of dopaminergic neurons in the substantia nigra, resulting in a variety of motor impairments (e.g., tremor, rigidity, bradykinesia), cognitive deficits (e.g., executive dysfunction, dementia), and psychological symptoms [17]. Like HD, roughly 50% of those with PD are estimated to have anxiety and/or depression [6,18]; thus, both populations may benefit from psychiatric interventions for improving quality of life [19,20]. Despite their different etiologies, comparisons between PD and HD can highlight the unique and shared burdens of these conditions and inform the design of interventions to effectively offset these burdens to both patients and care partners. These comparisons also may inform resource allocation and inform clinical care teams on how to better advise and prepare patients and their families for the sequelae associated with HD.

In the absence of effective disease-modifying or curative therapies and interventions for HD [21,22], efforts to improve health-related quality of life (HRQoL) and mental health must focus on actionable, real-world targets. Here, we aimed to evaluate the humanistic and clinical burdens reported among manifest individuals with HD and HD care partners, including the occurrence of comorbid mental health conditions, their severity, and overall impacts on HRQoL. These results are contextualized against adults and care partners selected from two relevant populations: the broader, non-neurodegenerative General Population, and a second neurodegenerative population (Parkinson’s Disease). The aim is to identify the shared as well as unique or incremental burdens that are experienced by those with HD as compared to those with Parkinson’s Disease or the General Population, thereby informing burden-modifying approaches for management of HD.

## 2. Methods

### 2.1. Study Design

This cross-sectional study was conducted using data obtained from the National Health and Wellness Survey (NHWS, Kantar Health, New York, NY, USA) [23,24,25] and the Rare Patient Voice (patient panel) [26]. Primary data from HD patients and care partners was collected via a customized survey administered by the Rare Patient Voice (July 2019–August 2019), which included sociodemographic characteristics, general health, HRQoL, and comorbid diagnoses (e.g., anxiety, depression). Secondary data from the 2018 NHWS survey was obtained to provide comparison cohorts which allowed us to examine the relative burdens associated with HD versus Parkinson’s Disease (PD) or the General Population (GP) (see Study Population for details on each cohort). The NHWS is a nationally representative, online survey conducted annually in the US, which queries over 200 health conditions, including questions pertaining to HRQoL, interactions with healthcare providers (e.g., physicians, physician assistants, and nurse practitioners), general health behaviors (e.g., smoking, exercising, BMI), and sociodemographic characteristics. All research reported here was reviewed and granted exemption by the Pearl Institutional Review Board (Indianapolis, IN, USA) and conducted in accordance with the Good Pharmacoepidemiology Practices (GPP) guidelines issued by the International Society for Pharmacoepidemiology (ISPE).

### 2.2. Study Population

HD Patients. Adults aged 18–70 years at the time of the survey who self-reported a diagnosis of HD were eligible to participate, provided they understood English and agreed to the informed consent. Individuals with juvenile HD and/or those who did not report experiencing at least one of the motor disruptions typical of HD (i.e., balance or gait issues, rigidity, chorea/dystonia, slowed eye movement, difficulty speaking or swallowing) were excluded from participation. These restrictions ensured that study conclusions were isolated to manifest individuals with HD. Consented patients are hereafter referred to as “individuals with HD” (IHD, n = 41).

HD Care Partners. Adults aged 18–70 years at the time of survey who devoted ≥5 h/week to the care of an adult family member with HD (i.e., reported being involved in their daily activities, emotional support, treatment decisions, and/or accompanied them to medical appointments) were eligible to participate, provided that they understood English and agreed to informed consent. Individuals < 18 years of age, those who were paid professional care takers, and/or did not meet the minimum weekly care requirements were excluded from participation. These restrictions ensured that all care partners were family members as opposed to paid employees; care partners who were reimbursed for their duties by the government or their insurance agency remained eligible to participate. Consented HD care partners are hereafter referred to as “HD care partners” (HD-CP, n = 80).

Comparison Cohorts. In order to contextualize the experiences of IHDs and HD-CPs, relevant cohorts were selected from the NHWS for comparison against the HD cohorts: (a) Individuals who self-reported a diagnosis of Parkinson’s Disease (IPD, n = 118) or who reported caring for an adult with Parkinson’s Disease (PD-CP, n = 385) enabled a specific comparison of the shared and divergent burdens of HD relative to PD, a separate neurodegenerative motor disorder; (b) Individuals without PD or HD (GP, n = 123) or who reported caring for a patient without PD or HD (GP-CP, n = 240) enabled us to further contrast these burdens relative to the broader General Population.

### 2.3. Outcome Measures

The results reported here represent a subset of total data collected about the humanistic, economic, and clinical burdens associated with HD. A full description of all variables collected can be found in the Supplementary Materials (Table S1). Here, we focus on describing the humanistic and clinical outcomes collected during our study. Unless otherwise stated, variables listed were compared between HD, PD, and GP (i.e., patient) cohorts separate from HD-CP, PD-CP, and GP-CP (i.e., care partner) cohorts.

Demographics and Health Characteristics. Demographics collected in each cohort included age, gender, employment status, household income, and US geographic region. Additional health characteristics collected among the HD cohorts included symptomology over the past 12 months and disease stage (Early-Stage (Stages 1 and 2); Mid-Stage (Stage 3); Late-Stage (Stages 4 and 5)) [27]. Per the Huntington’s Disease Society of America (HDSA), patients in the Early-Stage are the most independent with mild symptom manifestation, those in Mid-Stage begin to require external supervision and/or assistance as they manage increasingly severe symptoms (e.g., chorea), and those in Late-Stage experience the most severe symptoms and thus require assistance in all activities of daily living [27]. Complementary to their self-reported staging and symptoms, HD patients and care partners also rated their current overall physical, mental, emotional, financial, and social “health” each on a 5-point Likert scale (1 = Poor, 5 = Excellent).

Health-Related Quality of Life. General HRQoL was evaluated with the EuroQol 5-dimension health questionnaire (EQ-5D-5L), a widely used scale designed to assess 5 dimensions of general health (mobility, self-care, usual activities, pain/discomfort, and anxiety/depression). The score ranges from 0 to 1, with 0 being equal to death and 1 being equal to full health. The EuroQol Visual Analogue Scale (EQ-VAS; score: 0 to 100) consists of a line on which respondents indicate their self-rated health, with the endpoints of the line being best imaginable health state (score = 100) and worst imaginable health state (score = 0) [28].

Comorbidity Burden. Comorbidity burden was evaluated by probing patients and care partners to report up to 20 diagnosed conditions, including anxiety, autoimmune diseases, bipolar disorder, depression, cancer, diabetes, and heart disease. In addition to a self-reported diagnosis of depression, mental health was further probed by administering the Patient Health Questionnaire (PHQ-9). The PHQ-9 is a validated instrument that queries about depressive symptoms experienced in the past 2 weeks, with items scored on a 0 (Not at all) to 3 (Nearly every day) scale. Scores of 5, 10, and 20 serve as cut-offs indicating mild, moderate, and severe levels of depression [29].

Perceived Impacts of HD. In addition to direct impacts on health and HRQoL, respondents were asked about the subjective impacts of HD on their lives. Questions probed on specific emotional impacts (e.g., isolation, relationships, self-esteem), as well as functional and/or physical impacts (e.g., fears about declining health, loss of independence, and disruptions to work and home life) associated with HD. These results are summarized descriptively/graphically for each of the HD and HD-CP cohorts. See Supplementary Materials for additional details (Tables S2 and S3).

### 2.4. Statistical Analyses

Patient (IHD, IPD, GP) and care partner (HD-CP, PD-CP, GP-CP) cohorts were compared in separate analyses using t-tests (for continuous variables) and chi-square tests (for categorical variables); in both cases, analyses focused on comparing the respective HD cohorts to PD and GP cohorts (no direct comparisons between PD and GP cohorts were conducted). These results revealed baseline demographic differences (by age and sex) between the HD, PD, and GP cohorts which could confound interpretation of study outcomes; thus, follow-up analyses were conducted adjusting for age and/or sex.

For comparisons between HD and PD cohorts, results were age- and/or sex-adjusted via multivariable models. For comparisons between HD and GP cohorts, results were age- and/or sex-adjusted via propensity-score matching at a 1:3 ratio using recommended methods described elsewhere [30,31,32]. The decision to use multivariable modelling versus propensity score matching for the adjustment was determined by sample sizes (the much larger GP cohorts enabled the use of propensity matching, which was not possible due to the small sample sizes associated with the PD cohorts, thereby necessitating multivariable adjustment).

Unless otherwise stated, results are presented as means ± standard deviation (M ± SD) and a significance level of <0.05 was interpreted as statistically significant. Normally distributed outcomes (e.g., EQ-5D) were predicted using generalized linear models (GLMs) with normal distributions and identity link functions; outcomes with skewed distributions (e.g., PHQ-9) were predicted using GLMs with negative binomial distributions and log-link functions. A Poisson regression was fit for count data (e.g., comorbidities). All statistical analyses were conducted using SPSS version 23.

## 3. Results

### 3.1. Demographic Characteristics

As shown in Table 1, IHD were significantly younger (45.61 ± 12.52 vs. 58.17 ± 16.03 years; *p* < 0.001) and more likely to be female (68.3% vs. 38.1%; *p* = 0.01) than IPD; these results are consistent with the disease characteristics of PD and HD that have been described elsewhere [7,33,34]. While unemployment rates were similar (70.8% vs. 67.8%), roughly half of the IPD sample (41.5%) earned between $50–100 k, whereas the majority of IHD (68.3%) earned less than $50 k (NS). IHD respondents did not differ from the matched GP on any of these demographic criteria (all NS).

Apart from a higher representation of women (81.3% vs. 65.5%; *p* < 0.001), HD-CP resembled the characteristics of the PD-CP cohort (all NS). No significant differences were observed between HD-CP and the matched GP-CP.

### 3.2. Health Characteristics

As shown in Table 2, the majority of the IHD cohort had Early- or Mid-Stage HD (87.8%) and were utilizing a care partner (65.9%), whereas the majority of HD-CP reported caring for adults with Mid- or Late-Stage HD (91.2%). The most reported HD symptoms experienced in the past 12 months among IHD were: difficulty in remembering (70.7%), difficulty in planning/problem solving/decision making (70.7%), and involuntary movements or spasms (63.4%).

Physical, emotional, and social health were reported as poor/fair in nearly half of IHD (46.3%, 46.4% and 43.9%, respectively) and in roughly one-third of HD-CP (21.3%, 36.6%, and 28.8%, respectively). Additionally, more than half of IHD perceived their overall mental and financial health as poor/fair (58.5% and 68.2%), while 16.3% and 31.3% of HD-CP reported their own mental and financial health as poor/fair.

### 3.3. Perceived Impacts of HD

**Figure 1:**
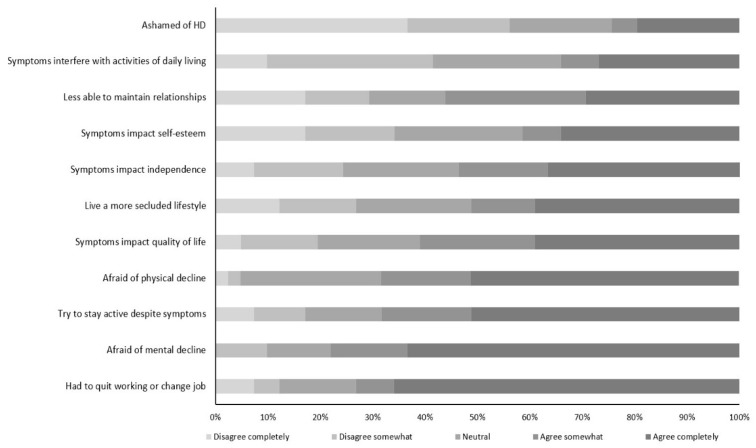
Impact of HD on individuals with HD. HD, Huntington’s disease. Unadjusted data.

As shown in Figure 1 (see also Table S2), more than half of IHD agreed somewhat/completely that HD had a significant impact on their overall quality of life (61.0%), ability to maintain relationships (56.1%), and independence (53.7%). Approximately three-quarters of individuals agreed somewhat/completely that their HD symptoms caused them to change jobs or quit working (73.2%) and that they most feared the eventual mental decline (78.0%) or physical decline (68.3%) associated with HD.

**Figure 2:**
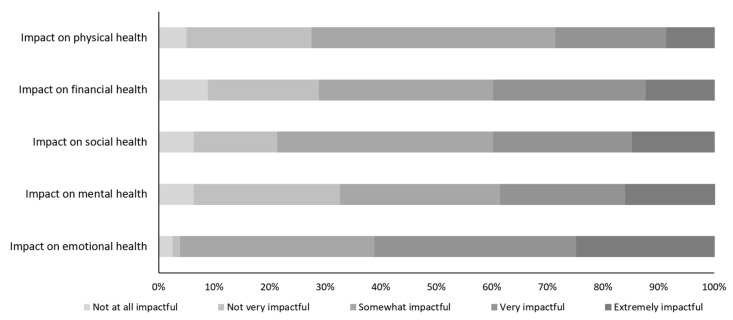
Impact of HD on care partners. Unadjusted data.

As shown in Figure 2 (see also Table S3), more than half of HD-CP reported that caring for a patient with HD was very/extremely impactful on their emotional health (61.3%) and required them to take time off from their work or studies (62.6%). Many HD-CP reported that they could not dedicate as much time as they would like to their job (69.2%) or could not focus on work (46.2%). In HD-CP receiving an education, roughly one-third reported struggling to catch up (36.4%) or having to withdraw or drop out of school (27.3%) due to caregiving responsibilities. Almost half of HD-CP reported feeling lonely/isolated (45.0%) or having lost some friends (35.0%). The majority reported a negative impact of caregiving on their future plans (63.8%), with difficulty saving money (60.8%), balancing a career (41.2%), and changing residence (39.2%) as limiting factors.

### 3.4. Comorbidity Burden and Health-Related Quality of Life

**Figure 3:**
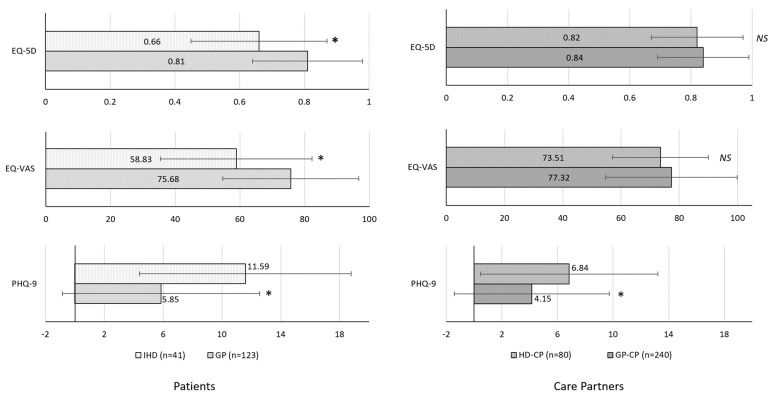
HRQoL outcomes of HD relative to GP. All comparisons adjusted for age and sex using propensity-score matching. Error bars indicate standard deviation. * *p* < 0.001. GP, general population; IHD, individuals with Huntington’s disease; CP, care partner; HRQoL, health-related quality of life.

As shown in Figure 3, IHD had consistently lower HRQoL relative to the GP, with significant differences observed for EQ-5D (0.66 ± 0.21 vs. 0.81 ± 0.17) and EQ-VAS scores (58.83 ± 23.43 vs. 75.68 ± 21.00) (all *p* < 0.001).

**Figure 4:**
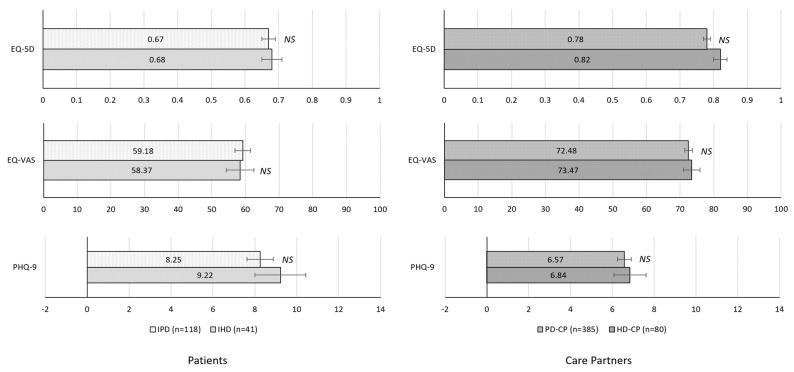
HRQoL outcomes of HD relative to PD. IHD vs. IPD comparisons adjusted for sex using multivariable regression; HD-CP vs. PD-CP comparisons adjusted for sex and age using multivariable regression. Error bars indicate standard error of the mean. IPD, individuals with Parkinson’s Disease; IHD, individuals with Huntington’s disease; CP, care partner; HRQoL, health-related quality of life.

Likewise, PHQ-9 scores were significantly higher in IHD than GP, suggesting greater severity of depression (11.59 ± 7.20 vs. 5.85 ± 6.71; *p* < 0.001). Among care partners, HD-CP demonstrated significantly greater depression than GP-CP based on PHQ-9 scores (6.84 ± 6.38 vs. 4.15 ± 5.58; *p* < 0.001) but similar levels of HRQoL (EQ-5D and EQ-VAS, both NS). Notably, no significant differences were observed between HD and PD cohorts (both patients and care partners) on any of these measures (see Figure 4, all NS).

**Figure 5:**
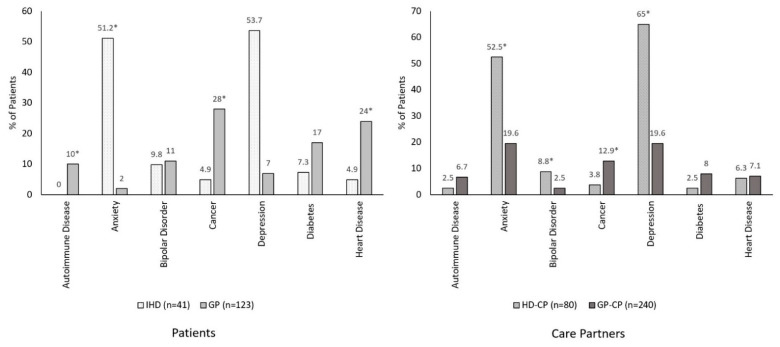
Comorbidities among HD relative to GP. All comparisons adjusted for age and sex using propensity-score matching. * *p* < 0.05. CP, care partner; GP, general population; IHD, individuals with Huntington’s disease.

**Figure 6:**
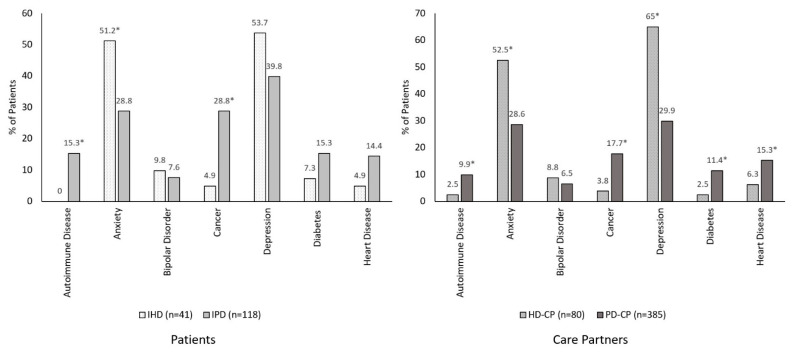
Comorbidities among HD relative to PD. IHD vs. IPD comparisons adjusted for sex using multivariable regression; HD-CP vs. PD-CP comparisons adjusted for sex and age using multivariable regression. * *p* < 0.05. CP, care partner; IHD, individuals with Huntington’s disease; IPD, individuals with Parkinson’s disease.

As shown in Figure 5 and Figure 6, comorbidities varied considerably among HD, PD, and GP cohorts. Among patients, IHD was associated with higher rates of anxiety (51.2% vs. 2.0%) and depression (53.7% vs. 7.0%), and lower rates of autoimmune disease (0% vs. 10.0%), cancer (4.9% vs. 28%), and heart disease (4.9% vs. 24%), relative to GP (Figure 5, all *p* < 0.05). Conversely, IHD was associated with higher rates of anxiety (51.2% vs. 28.8%) and lower rates of cancer (4.9% vs. 28.8%), relative to IPD (Figure 6, all *p* < 0.05).

Among care partners, HD-CP exhibited higher rates of anxiety (52.5% vs. 19.6%), bipolar disorder (8.8% vs. 2.5%), and depression (65.0% vs. 19.6%), and lower rates of cancer (3.8% vs. 12.9%), relative to GP-CP (Figure 5, all *p* < 0.05). Compared with PD-CP, HD-CP exhibited higher rates of anxiety (52.5% vs. 28.6%) and depression (65.0% vs. 29.9%), but lower rates of cancer (3.8% vs. 17.7%), diabetes (2.5% vs. 11.4), and autoimmune disease (2.5% vs. 9.9%) (Figure 6, all *p* < 0.05).

## 4. Discussion

Few studies have assessed the burden of IHD and HD-CPs relative to those without HD (i.e., GP) or to those with a different progressive neurological disease. Here, we examined the clinical and humanistic burdens of HD in this broader context by comparing to age- and/or sex-adjusted GP and PD cohorts. Our data support the evidence of a high burden for IHD and their care partners relative to GP (but similar burden as PD) and provide evidence of unique and common burdens observed among HD and PD cohorts.

Consistent with prior studies [35,36,37,38], IHD reported significantly poorer HRQoL than individuals without HD (i.e., GP), with lower scores being observed on both the EQ-5D and EQ-VAS. This result was not surprising, given the known clinical and symptomatic features of HD and their progressive role in disrupting one’s independence, capacity to work, and ability to fulfill routine activities of daily life [27]. Indeed, roughly 88% of our IHD cohort identified as being in the Early- to Mid-Stage of HD progression, with the most common symptoms being involuntary movements/spasms and cognitive issues. This finding is consistent with prior work implicating cognitive and motor impairment as some of the most impactful symptoms reported by patients and care partners [22], and as significant moderators of functional ability and HRQoL [14,39].

In addition to cognitive impairment, researchers have noted a substantial role of mental health and mood dysregulation in HD [5]. Prior studies have found depression and anxiety to be significant determinants of HRQoL in HD [15,37,40] and HD-CP [40]; in fact, anxiety and depression appear to be the most severely altered dimensions on the EQ-5D among those with HD [35]. Consistent with these results, IHD in this study exhibited approximately 2-fold higher prevalence of mental health-related disorders and more severe depression (as assessed by the PHQ-9) than GP—notably, the IHD cohort exceeded the diagnostic cut-off (PHQ-9 score of 10+) for major depressive disorder (PHQ-9: IHD = 11.6 vs. GP = 5.9) [29]. HD-CP also exhibited greater comorbid anxiety and depression, and more severe depression (as calculated by PHQ-9 scores) than GP-CP. These clinical findings are supported by respondents’ survey data, with half of HD-CP reporting feeling lonely/isolated and experiencing extreme impacts on emotional health. Similarly, over half of IHD report difficulty maintaining relationships, remaining independent, and being fearful of their eventual mental and physical decline. All these features may contribute to the greater degree of anxiety and depression observed among HD cohorts in this study.

Notably, there were a few key differences observed among IHD and HD-CP cohorts relative to IPD and PD-CP cohorts in this study—HD cohorts were roughly twice as likely to report having comorbid anxiety (IHD = 51.2% vs. IPD = 28.8%; HD-CP = 52.5% vs. PD-CP = 28.6%) and depression (HD-CP = 65.0% vs. PD-CP = 29.9%) than PD cohorts, despite reporting comparable levels of HRQoL (EQ-5D measures, NS). These results are consistent with a recent study that reported a higher incidence of anxiety and major depressive disorder in IHD than IPD, as well as the general population [41].

Given that both HD and PD are progressive neurological disorders with similar symptomatology, it is interesting to consider whether HD is specifically associated with greater mood dysregulation than PD, or whether this is a symptom of design artifact (e.g., sociodemographic differences that could contribute to underlying stress, e.g., financial stability). For example, IHD were significantly younger and tended to earn lower wages than IPD in this study—factors which could exacerbate stress in the face of financial concerns. Indeed, roughly three-quarters of our IHD cohort reported that their symptoms caused them to change jobs or quit working. The majority of HD-CP similarly noted that their work life had suffered because of caregiving and that they had difficulty saving money, in line with previous findings [42]. This consideration aside, previous research has suggested that IHD and HD-CP have greater mood disturbances than individuals with other progressive neurological illnesses, including individuals with IPD and multiple sclerosis (MS), and their care partners, respectively [15]. Others have implicated psychiatric disorders as an integral component of HD progression [43,44], which could explain to a certain extent the higher prevalence of depression and anxiety in IHD.

Considering the (1) high prevalence of anxiety and depression observed among HD and PD, (2) the impact of these psychiatric conditions on HRQoL, and (3) the carryover effects of these psychiatric symptoms on care partners, one would postulate that one of the most immediate ways to improve the lives of patients and care partners is by employing mental health interventions. Strikingly, however, there is a relative paucity of studies investigating psychological interventions among patients with these neurodegenerative diseases [19,20]. The limited evidence available suggests a role for CBT in the treatment of depression in PD and HD, but little is known about how to manage anxiety, and the available evidence is extremely limited in terms of methodology and outcome assessment [19,20,45]. Studies examining interventions for depression and anxiety among those with HD is especially lacking [45], partly due to a need for validated screening tools in these populations, for whom apathy, unawareness, and denial of symptoms can complicate accurate measurement [1,46]. Together, our findings emphasize the importance of psychological interventions and access to mental health services as a pivotal role in offsetting disease burden among the HD community.

### Strengths and Limitations

Though the study is comprehensive and has detailed analysis, it has a few limitations. First, data collected was self-reported and could not be independently verified through other data sources; thus, a possibility of recall bias might exist. For example, anosognosia (unawareness of one’s deficits) is estimated to impact between 25–50% of those with HD [47,48,49], which may have contributed to under-reporting of complaints or concerns from HD patients; thus, the true magnitude of effect between HD and GP may be greater than estimated in this study. Second, individuals with HD and HD-CP were recruited from the Rare Patient Voice panel, which might not be representative of the overall HD population. For example, individuals with HD with limited internet access might be underrepresented in our sample, and individuals with greater disease severity might have opted against completing the online survey due to physical and/or cognitive impairment. While we attempted to minimize these potential biases by providing patients with the option of having a care partner assisting (or conducting a telephone-assisted interview in lieu of an online survey), we cannot rule out that those with the greatest burden (i.e., most severe forms of any disease, including HD and PD) may be under-represented in this study. That said, any such biases would not account for the differences observed here (i.e., the inclusion of more severe patients would only have only increased the magnitude of effect reported). Third, the low sample size of the study (specifically in regard to rare patient populations, like those with PD) should be taken into account when interpreting the study results. Fourth, the current study was cross-functional in design, thus causality could not be determined. Finally, while matching and multivariable regressions were conducted to account for imbalances in age and sex distributions between the cohorts, there remains a potential for residual confounding by variables not accounted for in our analyses.

## 5. Conclusions

While care management services are an integral part of healthcare service delivery in HD [50,51,52], studies suggest that healthcare and social support needs for HD and HD-CP remain largely unmet [53,54]. It is therefore, as with other neurologic conditions [55], important to increase awareness among providers and payers regarding the needs of the HD community to improve quality and continuity of care, which in turn may alleviate the burden associated with HD. Here, we demonstrate that IHD and HD-CP experience a significantly greater clinical and humanistic burden compared to the general population, and nearly twice the mental health comorbidity reported by IPD and PD-CP. These results highlight the need for ensuring provision of adequate mental health and preventive services, and the need for effective therapies that prevent or delay disease progression, which may reduce the burden of HD and improve HRQoL of diagnosed individuals and their care partners.

## Figures and Tables

**Table 1:** Demographic characteristics of all cohorts.

		Individuals/Patients			Care Partners		
		IHD (n = 41)	IPD (NHWS) (n = 118)	Matched GP (NHWS) (n = 123)	HD-CP (n = 80)	PD-CP (NHWS) (n = 385)	Matched GP-CP (NHWS) (n = 240)
Age (Mean ± SD)		45.61 ± 12.52	58.17 ± 16.03 **	45.61 ± 12.41	46.80 ± 15.06	48.02 ± 16.81	46.80 ± 15.00
Gender, n (%)	Female	28 (68.3)	45 (38.1) **	84 (68.3)	65 (81.3)	252 (65.5) **	195 (81.3)
Current employment status, n (%)	Non-employed	29 (70.7)	80 (67.8)	38 (30.9) **	25 (31.3)	169 (43.9) *	96 (40.0)
	Employed (working for pay)	12 (29.3)	38 (32.2)	85 (69.1)	55 (68.8)	216 (56.1)	144 (60.0)
Own household income (grouped), n (%)	Less than $50,000	28 (68.3)	44 (37.3) *	44 (35.8) **	34 (42.5)	152 (39.5)	97 (40.4)
	$50,000 to less than $100,000	9 (22.0)	49 (41.5)	43 (35.0)	27 (33.8)	106 (27.5)	79 (32.9)
	$100,000 to less than $150,000	2 (4.9)	14 (11.9)	19 (15.4)	12 (15.0)	71 (18.4)	33 (13.8)
	$150,000 or more	2 (4.9)	7 (5.9)	12 (9.8)	3 (3.8)	34 (8.8)	17 (7.1)
	Prefer not to answer	0 (0.0)	4 (3.4)	5 (4.1)	4 (5.0)	22 (5.7)	14 (5.8)
Region, n (%)	Northeast	6 (14.6)	15 (12.7) *	26 (21.1) *	15 (18.8)	75 (19.5)	58 (24.2)
	North Central	9 (22.0)	27 (22.9)	23 (18.7)	22 (27.5)	87 (22.6)	53 (22.1)
	South	15 (36.6)	43 (36.4)	43 (35.0)	31 (38.8)	129 (33.5)	81 (33.8)
	West	11 (26.8)	33 (28.0)	31 (25.2)	12 (15.0)	94 (24.4)	48 (20.0)

Omnibus *p*-value indicated as * *p* < 0.01; ** *p* < 0.05; all statistics reported relative to HD or HD-CP cohorts, for patients and care partner analyses, respectively. IHD, individuals with Huntington’s disease; IPD, individuals with Parkinson’s disease; GP, general population; HD-CP, Huntington’s disease care partners; IPD-CP, Parkinson’s disease care partners; GP-CP, general population care partners; NHWS, National Health and Wellness Survey; SD, standard deviation.

**Table 2:** Health characteristics of individuals with HD and care partners.

		IHD (n = 41)	HD-CP (n = 80)
Disease stage *, n (%)	Early-Stage	20 (48.8)	7 (8.8)
	Mid-Stage	16 (39.0)	46 (57.5)
	Late-Stage	5 (12.2)	27 (33.8)
Current overall health			
Physical health, n (%)	Poor	6 (14.6)	2 (2.5)
	Fair	13 (31.7)	15 (18.8)
	Good	14 (34.1)	31 (38.8)
	Very Good	8 (19.5)	27 (33.8)
	Excellent	0 (0.0)	5 (6.3)
Mental health, n (%)	Poor	6 (14.6)	0 (0.0)
	Fair	18 (43.9)	13 (16.3)
	Good	10 (24.4)	27 (33.8)
	Very Good	5 (12.2)	24 (30.0)
	Excellent	2 (4.9)	16 (20.0)
Emotional health, n (%)	Poor	7 (17.1)	3 (3.8)
	Fair	12 (29.3)	26 (32.5)
	Good	15 (36.6)	25 (31.3)
	Very Good	5 (12.2)	20 (25.0)
	Excellent	2 (4.9)	6 (7.5)
Financial health, n (%)	Poor	14 (34.1)	11 (13.8)
	Fair	14 (34.1)	14 (17.5)
	Good	8 (19.5)	28 (35.0)
	Very Good	5 (12.2)	17 (21.3)
	Excellent	0 (0.0)	10 (12.5)
Social health, n (%)	Poor	2 (4.9)	5 (6.3)
	Fair	16 (39.0)	18 (22.5)
	Good	13 (31.7)	21 (26.3)
	Very Good	8 (19.5)	21 (26.3)
	Excellent	2 (4.9)	15 (18.8)
HD Symptomology			
Symptoms experienced in the past 12 months, n (%)	Difficulty walking or balancing/falling	21 (51.2)	NA
	Stiffness in the body	14 (34.1)	NA
	Having trouble grasping small items such as using a pen, hand sewing, etc.	24 (58.5)	NA
	Difficulty speaking	20 (48.8)	NA
	Difficulty swallowing	22 (53.7)	NA
	Involuntary movements or spasms in the body	26 (63.4)	NA
	Muscle contractions that change the shape of the body or make it difficult to stand up straight	16 (39)	NA
	Slowed eye movement	15 (36.6)	NA
	Difficulty remembering	29 (70.7)	NA
	Difficulty with planning, problem solving and/or decision marking	29 (70.7)	NA
	Feeling very sad or depressed	21 (51.2)	NA
	Easily becoming angry or annoyed	22 (53.7)	NA
	Loss of muscle strength due to lack of physical activity	16 (39.0)	NA
	Unable to resist a temptation, urge or impulse	20 (48.8)	NA
	Inability to control a sudden desire to say or do something	16 (39.0%)	NA
	None of the above/Don’t know	0 (0)	NA
Living status			
HD has a care partner, n (%)	Yes, I have a care partner	27 (65.9)	NA
	No, I do not have a care partner	14 (34.1)	NA

* Data for individuals represent their own disease stage and data for care partners represent the disease stage of the individuals they are taking care of. IHD, individuals with Huntington’s disease; HD-CP, Huntington’s disease care partners; NHWS, National Health and Wellness Survey; NA, not applicable; SD, standard deviation.

## Data Availability

The data that support the findings of this study were used under license by Cerner Enviza, and so are not publicly available. However, data can be made available for non-commercial use from the authors upon reasonable request and with the explicit written permission of Cerner Enviza.

## Supplementary Materials

The following supporting information can be downloaded at: https://www.mdpi.com/article/10.3390/brainsci12020161/s1, Table S1: Overview of survey data collected; Table S2: Impact of HD on patients; Table S3: Impact of HD on care partners.

## References

[1] Bates G.P., Dorsey R., Gusella J.F., Hayden M.R., Kay C., Leavitt B.R., Nance M., Ross C.A., Scahill R.I., Wetzel R. (2015). Huntington disease. Nat. Rev. Dis. Primers.

[2] Huntington’s Disease Society of America. https://Hdsa.Org/What-Is-Hd/Overview-of-Huntingtons-Disease/.

[3] Baig S.S., Strong M., Quarrell O.W. (2016). The global prevalence of Huntington’s disease: A systematic review and discussion. Neurodegener. Dis. Manag..

[4] MacDonald M.E. (1993). A novel gene containing a trinucleotide repeat that is expanded and unstable on Huntington’s disease chromosomes. The Huntington’s Disease Collaborative Research Group. Cell.

[5] Gubert C., Renoir T., Hannan A.J. (2020). Why Woody got the blues: The neurobiology of depression in Huntington’s disease. Neurobiol. Dis..

[6] Novak M.J.U., Tabrizi S.J. (2011). Huntington’s disease: Clinical presentation and treatment. Int. Rev. Neurobiol..

[7] Myers R.H. (2004). Huntington’s disease genetics. NeuroRx.

[8] Pickett T., Altmaier E., Paulsen J.S. (2007). Caregiver burden in Huntington’s disease. Rehabil. Psychol..

[9] Paulsen J.S., Nehl C., Hoth K.F., Kanz J.E., Benjamin M., Conybeare R., McDowell B., Turner B. (2005). Depression and stages of Huntington’s disease. J. Neuropsychiatry Clin. Neurosci..

[10] Paulsen J.S., Hoth K.F., Nehl C., Stierman L. (2005). Critical periods of suicide risk in Huntington’s disease. Am. J. Psychiatry.

[11] Dorsey E.R., Huntington Study Group COHORT Investigators (2012). Characterization of a large group of individuals with Huntington disease and their relatives enrolled in the COHORT study. PLoS ONE.

[12] Kirkwood S.C., Su J.L., Conneally P., Foroud T. (2001). Progression of symptoms in the early and middle stages of Huntington disease. Arch. Neurol..

[13] Yu M., Tan K., Koloms K., Bega D. (2019). Assessment of caregiver burden in Huntington’s disease. J. Huntington’s Dis..

[14] Ready R.E., Mathews M., Leserman A., Paulsen J.S. (2008). Patient and caregiver quality of life in Huntington’s disease. Mov. Disord..

[15] McCabe M.P., Firth L., O’Connor E. (2009). Mood and quality of life among people with progressive neurological illnesses. Int. J. Clin. Health Psychol..

[16] Zhang Z.X., Román G.C. (1993). Worldwide occurrence of Parkinson’s disease: An updated review. Neuroepidemiology.

[17] Poewe W., Seppi K., Tanner C.M., Halliday G.M., Brundin P., Volkmann J., Schrag A.-E., Lang A.E. (2017). Parkinson disease. Nat. Rev. Dis. Primers.

[18] Yamanishi T., Tachibana H., Oguru M., Matsui K., Toda K., Okuda B., Oka N. (2013). Anxiety and depression in patients with Parkinson’s disease. Intern. Med..

[19] Zarotti N., Eccles F.J.R., Foley J.A., Paget A., Gunn S., Leroi I., Simpson J. (2021). Psychological interventions for people with Parkinson’s disease in the early 2020s: Where do we stand?. Psychol. Psychother..

[20] Zarotti N., Dale M., Eccles F., Simpson J. (2020). Psychological Interventions for People with Huntington’s Disease: A Call to Arms. J. Huntington’s Dis..

[21] Frank S. (2014). Treatment of Huntington’s disease. Neurotherapeutics.

[22] Simpson J.A., Lovecky D., Kogan J., Vetter L.A., Yohrling G.J. (2016). Survey of the Huntington’s disease patient and caregiver community reveals most impactful symptoms and treatment needs. J. Huntington’s Dis..

[23] Unni E.J., Sternbach N., Goren A. (2019). Using the Medication Adherence Reasons Scale (MAR-Scale) to identify the reasons for non-adherence across multiple disease conditions. Patient Prefer. Adherence.

[24] Nakata K., Tsuji T., Vietri J., Jaffe D.H. (2018). Work impairment, osteoarthritis, and health-related quality of life among employees in Japan. Health Qual. Life Outcomes.

[25] DiBonaventura M., Sun S.X., Bolge S.C., Wagner J.-S., Mody R. (2011). Health-related quality of life, work productivity and health care resource use associated with constipation predominant irritable bowel syndrome. Curr. Med. Res. Opin..

[26] Becker D.A., Long L., Santilli N., Babrowicz J., Peck E.Y. (2021). Patient, caregiver, and healthcare professional perspectives on seizure control and treatment goals. Epilepsy Behav..

[27] Huntington’s Disease Stages Huntington’s Disease Society of America. https://hdsa.org/what-is-hd/huntingtons-disease-stages/.

[28] Herdman M., Gudex C., Lloyd A., Janssen M., Kind P., Parkin D., Bonsel G., Badia X. (2011). Development and preliminary testing of the new five-level version of EQ-5D (EQ-5D-5L). Qual. Life Res..

[29] Manea L., Gilbody S., McMillan D. (2012). Optimal cut-off score for diagnosing depression with the Patient Health Questionnaire (PHQ-9): A meta-analysis. Can. Med. Assoc. J..

[30] Rassen J.A., Shelat A.A., Myers J., Glynn R.J., Rothman K.J., Schneeweiss S. (2012). One-to-many propensity score matching in cohort studies. Pharmacoepidemiol. Drug Saf..

[31] Austin P.C. (2010). Statistical criteria for selecting the optimal number of untreated subjects matched to each treated subject when using many-to-one matching on the propensity score. Am. J. Epidemiol..

[32] Austin P.C. (2011). An Introduction to Propensity Score Methods for Reducing the Effects of Confounding in Observational Studies. Multivar. Behav. Res..

[33] Reekes T.H., Higginson C.I., Ledbetter C.R., Sathivadivel N., Zweig R.M., Disbrow E.A. (2020). Sex specific cognitive differences in Parkinson disease. npj Parkinson’s Dis..

[34] Pagano G., Ferrara N., Brooks D.J., Pavese N. (2016). Age at onset and Parkinson disease phenotype. Neurology.

[35] Dorey J., Clay E., Khemiri A., Belhadj A., Cubillo P.T., Toumi M. (2016). The quality of life of Spanish patients with Huntington’s disease measured with H-QoL-I and EQ-5D. J. Mark. Access Health Policy.

[36] Calvert M., Pall H., Hoppitt T., Eaton B., Savill E., Sackley C. (2013). Health-related quality of life and supportive care in patients with rare long-term neurological conditions. Qual. Life Res..

[37] Ho A.K., Gilbert A.S., Mason S.L., Goodman A.O., Barker R.A. (2009). Health-related quality of life in Huntington’s disease: Which factors matter most?. Mov. Disord..

[38] Ho A.K., Robbins A.O.G., Walters S.J., Kaptoge S., Sahakian B.J., Barker R.A. (2004). Health-related quality of life in Huntington’s disease: A comparison of two generic instruments, SF-36 and SIP. Mov. Disord..

[39] Zielonka D., Ren M., De Michele G., Roos R.A.C., Squitieri F., Bentivoglio A.R., Marcinkowski J.T., Landwehrmeyer G.B. (2018). The contribution of gender differences in motor, behavioral and cognitive features to functional capacity, independence and quality of life in patients with Huntington’s disease. Parkinsonism Relat. Disord..

[40] Banaszkiewicz K., Sitek E.J., Rudzińska M., Sołtan W., Sławek J., Szczudlik A. (2012). Huntington’s disease from the patient, caregiver and physician’s perspectives: Three sides of the same coin?. J. Neural Transm..

[41] Ishihara L., Wild E.J., Oliveri D. (2020). Comorbidities and Medications in Huntington’s Disease, Parkinson’s Disease and the General Population in a US Claims Database. Neurology.

[42] Raimundo K., Tan R., To T.M., Courcy J., Ondhia U., Rickards H., Martha N. (2020). Impact of caring for patients with Huntington’s disease on work status. Neurology.

[43] Paoli R.A., Botturi A., Ciammola A., Silani V., Prunas C., Lucchiari C., Zugno E., Caletti E. (2017). Neuropsychiatric Burden in Huntington’s Disease. Brain Sci..

[44] Pla P., Orvoen S., Saudou F., David D.J., Humbert S. (2014). Mood disorders in Huntington’s disease: From behavior to cellular and molecular mechanisms. Front. Behav. Neurosci..

[45] Ghielen I., Rutten S., Boeschoten R.E., Houniet-de Gier M., van Wegen E.E.H., van den Heuvel O.A., Cuijpers P. (2019). The effects of cognitive behavioral and mindfulness-based therapies on psychological distress in patients with multiple sclerosis, Parkinson’s disease and Huntington’s disease: Two meta-analyses. J. Psychosom. Res..

[46] Miyasaki J.M., Shannon K., Voon V., Ravina B., Kleiner-Fisman G., Anderson K., Shulman L.M., Gronseth G., Weiner W.J., Quality Standards Subcommittee of the American Academy of Neurology (2006). Practice Parameter: Evaluation and treatment of depression, psychosis, and dementia in Parkinson disease (an evidence-based review): Report of the Quality Standards Subcommittee of the American Academy of Neurology. Neurology.

[47] Isaacs D., Gibson J.S., Stovall J., Claassen D.O. (2020). The Impact of Anosognosia on Clinical and Patient-Reported Assessments of Psychiatric Symptoms in Huntington’s Disease. J. Huntington’s Dis..

[48] McCusker E., Loy C.T. (2014). The many facets of unawareness in huntington disease. Tremor Other Hyperkinet. Mov..

[49] Sitek E.J., Thompson J.C., Craufurd D., Snowden J.S. (2014). Unawareness of deficits in Huntington’s disease. J. Huntington’s Dis..

[50] Raimundo K., Tan R., To T.M., Courcy J., Ondhia U., Rickards H., Martha N. (2020). Patient and physician perspectives on the care and assistance needs in Huntington’s disease. Neurology.

[51] Veenhuizen R.B., Tibben A. (2009). Coordinated multidisciplinary care for Huntington’s disease. An outpatient department. Brain Res. Bull..

[52] Bourke D., Finucane G., Dysart J., Roxburgh R. (2012). The appointment of a Huntington’s disease nurse specialist has reduced admission rate and improved admission quality. J. Huntington’s Dis..

[53] Etchegary H. (2011). Healthcare experiences of families affected by Huntington disease: Need for improved care. Chronic Illn..

[54] Skirton H., Williams J.K., Jackson Barnette J., Paulsen J.S. (2010). Huntington disease: Families’ experiences of healthcare services. J. Adv. Nurs..

[55] Warshaw G.A., Bragg E.J. (2014). Preparing the health care workforce to care for adults with Alzheimer’s disease and related dementias. Health Aff..

