# Supplementary material for: Understanding the Burdens Associated with Huntington’s Disease in Manifest Patients and Care Partners–Comparing to Parkinson’s Disease and the General Population
Source: Brain Sci. 2022 Jan 26;12(2):161. doi: 10.3390/brainsci12020161 (PMC8869871; doi:10.3390/brainsci12020161)
Supplement: Supplementary file 1 [file brainsci-12-00161-s001.zip › brainsci-1541272-supplementary.pdf]

brain sciences

MDPI

Supplementary

Table S1. Overview of survey data collected.

Demographic Characteristics	IHD (on behalf of self)	HD-CP (on behalf of self)	HD-CP (on behalf of patient)
Age	X	X	X
Gender	X	X	X
Current employment status	X	X	X
Insurance	X	X	X
Prescription drug coverage	X	X	X
Annual household income	X	X	X
Current living situation (e.g., alone, in a nursing home, etc.)	X		X
US region of residence	X	X	
**Health Characteristics**			
Symptoms ever experienced	X		X
Symptoms experienced in the past 12 months	X		X
HD staging (Pre-, Early-, Mid-, Late-Stage)	X		X
Family member(s) diagnosed with Huntington's Disease	X	X	
Family member(s) with HD that you have cared for in past		X	
Body mass index (BMI)		X	
Overall physical health (1–5)	X	X	X
Overall mental health (1–5)	X	X	X
Overall emotional health (1–5)	X	X	X
Overall financial health (1–5)	X	X	X
Overall social health (1–5)	X	X	X
Diagnosed comorbidities (1–20)	X	X	X
Number of prescription medications taken per day (0–20)	X	X	X
Number of prescriptions taken for HD per day	X		X
Any prescriptions taken for HD in the past month (Y/N)	X		X
**Disease Management / HCRU**			
Time since first demonstrated motor symptoms	X		X
Time since diagnosis	X		X
Specialty of diagnosing physician	X		X
Occurrence (Y/N) of genetic testing (including CAG), pre/post diagnosis or symptom manifestation	X		X
CAG repeat number	X		X
Seen at specialty center of excellence	X		X
HCPs visited in past 12 months for HD	X		X
HCPs visited at HD specialty centers/centers of excellence	X		X
Primary healthcare provider for HD	X		X
Average frequency of HCP visits, per provider	X		X
Number of ER visits in the past 6 months	X	X	X
Number of hospitalizations in the past 6 months	X	X	X
Number of HCP visits in the past 6 months	X	X	X
Recency of last HCP visit		X	
**Assistance**			
Others involved in HD management	X		X
Hours that others spend on patient care per week	X		X
Type of unpaid help received, if any	X		X
Number of days using support services in past month, per service	X		X
Monthly out-of-pocket expenses for care, per service	X	X	
Total monthly out-of-pocket expenses for care	X	X	
Percentage of monthly income spent on out-of-pocket expenses	X	X	
Reasons for not using paid services		X	
Types of accommodations made to home/vehicles, if any	X	X	
Reasons for accommodations, aside from patient symptoms		X	
Perceived financial burden of out-of-pocket expenses (1–5)	X	X	
**Work and Activity Productivity Loss**			
Employed (Y/N)	X	X	
Absenteeism (absences from work due to own health)	X	X	
Presenteeism (lost productivity on the job due to own health)	X	X	
Overall work productivity impairment	X	X	

Activity impairment	X	X	
**Validated Scales**			
EQ5D score	X	X	
PHQ9 score	X	X	
HD PRO TRIAD score	X		
ACQLI score		X	
**Impact of HD on Patient**			
Emotional Impacts (1–5)			
Ability to maintain relationships	X		X
Live a more secluded lifestyle	X		X
Impacts self-esteem	X		X
Feel ashamed of HD	X		X
Functional Impacts (1–5)			
Afraid of mental decline	X		X
Quit working or changed job	X		X
Symptoms impact quality of life	X		X
Symptoms impact independence	X		X
Symptoms interfere with activities of daily living	X		X
Physical Impacts (1–5)			
Afraid of physical decline	X		X
Try to stay active despite symptoms	X		X
Emotional State (over the past two weeks) (1–5)			
Have trouble expressing emotions (i.e., apathy)	X		X
Hard to find motivation to get things done	X		X
I am interested in things	X		X
**Access to Social Security Benefits**			
Ever applied for SSDB (Y/N)	X		X
Outcome of first application for SSDB	X		X
Applied again after first denial (Y/N)	X		X
Who helped with the SSDB application, if any	X		X
Insurance coverage at time of SSDB application	X		X
**Impact of HD on Caregiver** (items asked only to caregivers)			
Time and effort associated with caring			
Length of time caring for this family member (months/years)		X	
Hours devoted to patient care per week		X	
Number of visits per week associated with caring		X	
Times per day that prescription is administered to family member		X	
Burden of administering daily medications (1–5)		X	
Commute time associated with caring (one way trip)		X	
Hours per day available for socializing/hobbies/exercise/free time		X	
Hours per day available for errands/housework/chores		X	
Days of leisure missed in past month due to caretaking		X	
Impact of caring on overall health			
Physical health (1–5)		X	
Mental health (1–5)		X	
Financial health (1–5)		X	
Social health (1–5)		X	
Emotional health (1–5)		X	
Impact of caretaking on personal life			
Had to take time off work/studies last year, due to caring (Y/N)		X	
Ways that caring has impacted work		X	
Ways that caring has impacted education		X	
Ways that caring has impacted future plans/life goals		X	
Ways that caring negatively impacted future plans, if any		X	
Ways that caring positively impacted future plans, if any		X	
Ways that HD has impacted relationship with family member (the HD patient)		X	
Ways that HD has impacted relationship(s) with others		X	
Impact of genetic testing on personal life			
Has undergone genetic testing to determine own HD risk (Y/N)		X	
Reason for electing for genetic testing		X	
Whether caring influenced decision to receive genetic testing<br>Ways that genetic testing has impacted relationship with family member (the HD patient)		X<br>X	
Ways that genetic testing has impacted relationship(s) with others		X	

Ways that being gene negative impacted relationships(s), if any		X	
Ways that genetic testing impacted future plans/life goals		X	
Ways that genetic testing negatively impacted future plans, if any		X	
Ways that genetic testing positively impacted future plans, if any		X	
Impact of genetic testing on overall health		X	
Physical health (1–5)		X	
Mental health (1–5)		X	
Financial health (1–5)		X	
Social health (1–5)		X	
Emotional health (1–5)		X	
Role of Long-Term Care			
Have considered placing HD family member in nursing home or long-term care facility (Y/N)		X	
How long did it take to decide to place HD family member in nursing home or long-term care facility (years/months)		X	
Length of time HD family member has been living in a nursing home or long-term care facility (years/months)		X	
Living arrangement, prior to long-term care		X	

ACQLI, Alzheimer's carer's quality of life inventory; BMI, body mass index; CAG, cytosine, adenine, and guanine; CP, care partner; HCP, health care professional; HD, Huntington's disease; EQ-5D, EuroQol 5-dimension; ER, emergency room; IHD, individuals with Huntington's disease; PHQ-9, patient health questionnaire; PRO, patient reported outcome; QoL, quality of life; SSDB, social security disability benefits.

Table S2. Impact of HD on patients

		**IHD (n = 41)**
**Social**		*n (%)*
**Impact on HD patient's ability to maintain relationships**	Disagree completely	7 (17.1)
	2	5 (12.2)
	3	6 (14.6)
	4	11 (26.8)
	Agree completely	12 (29.3)
**HD patient's more secluded lifestyle**	Disagree completely	5 (12.2)
	2	6 (14.6)
	3	9 (22.0)
	4	5 (12.2)
	Agree completely	16 (39.0)
**HD patient had to quit working or change job**	Disagree completely	3 (7.3)
	2	2 (4.9)
	3	6 (14.6)
	4	3 (7.3)
	Agree completely	27 (65.9)
**Emotional**		
**Impact on HD patient's self-esteem**	Disagree completely	7 (17.1)
	2	7 (17.1)
	3	10 (24.4)
	4	3 (7.3)
	Agree completely	14 (34.1)
**HD patient's feelings of shame**	Disagree completely	15 (36.6)
	2	8 (19.5)
	3	8 (19.5)
	4	2 (4.9)
	Agree completely	8 (19.5)
**HD patient's fear of mental decline**	Disagree completely	0 (0.0)
	2	4 (9.8)
	3	5 (12.2)
	4	6 (14.6)
	Agree completely	26 (63.4)
**Overall impact on HD patient's quality of life**	Disagree completely	2 (4.9)
	2	6 (14.6)
	3	8 (19.5)
	4	9 (22.0)
	Agree completely	16 (39.0)
**Impact on HD patient's independence**	Disagree completely	3 (7.3)
	2	7 (17.1)

	3	9 (22.0)
	4	7 (17.1)
	Agree completely	15 (36.6)
**HD patient's fear of physical decline**	Disagree completely	1 (2.4)
	2	1 (2.4)
	3	11 (26.8)
	4	7 (17.1)
	Agree completely	21 (51.2)
**HD patient's trouble expressing emotions**	Never, 1	8 (19.5)
	Rarely, 2	6 (14.6)
	Sometimes, 3	13 (31.7)
	Often, 4	13 (31.7)
	Always, 5	1 (2.4)
**HD patient's lack of motivation**	Never, 1	2 (4.9)
	Rarely, 2	4 (9.8)
	Sometimes, 3	11 (26.8)
	Often, 4	16 (39.0)
	Always, 5	8 (19.5)
**HD patient's interest in things**	Never, 1	1 (2.4)
	Rarely, 2	4 (9.8)
	Sometimes, 3	17 (41.5)
	Often, 4	11 (26.8)
	Always, 5	8 (19.5)
**Physical**		
**Impact on HD patient's ability to complete activities of daily living**	Disagree completely	4 (9.8)
	2	13 (31.7)
	3	10 (24.4)
	4	3 (7.3)
	Agree completely	11 (26.8)
**HD patient tries to stay active despite symptoms**	Disagree completely	3 (7.3)
	2	4 (9.8)
	3	6 (14.6)
	4	7 (17.1)
	Agree completely	21 (51.2)

HD, Huntington's disease; IHD, individuals with Huntington's disease.

Table S3. Impact of HD on care partners

		**HD-CP (n = 80)**
**Physical**		*n (%)*
**Impact on physical health**	Not at all impactful	4 (5.0)
	Not very impactful	18 (22.5)
	Somewhat impactful	35 (43.8)
	Very impactful	16 (20.0)
	Extremely impactful	7 (8.8)
**Financial**		
**Impact on financial health**	Not at all impactful	7 (8.8)
	Not very impactful	16 (20.0)
	Somewhat impactful	25 (31.3)
	Very impactful	22 (27.5)
	Extremely impactful	10 (12.5)
**Mental**		
**Impact on mental health**	Not at all impactful	5 (6.3)
	Not very impactful	21 (26.3)
	Somewhat impactful	23 (28.8)
	Very impactful	18 (22.5)
	Extremely Impactful	13 (16.3)
**Emotional**		
**Impact on emotional health**	Not at all impactful	2 (2.5)
	Not very impactful	1 (1.3)
	Somewhat impactful	28 (35.0)
	Very impactful	29 (36.3)
	Extremely impactful	20 (25.0)
**Impact on future plans/life**	It has had a positive impact on my future plans/life goals	14 (17.5)

	It has had a negative impact on my future plans/life goals	51 (63.8)
	It has had no impact on my future plans/life goals	15 (18.8)
**Negative impact on future plans**	Prevented me from having children	5 (9.8)
	Made it hard to save money for the future	31 (60.8)
	Made having a career harder	21 (41.2)
	Prevented me from moving to a different city or place	20 (39.2)
	Prevented me from getting a better education	7 (13.7)
	Prevented me from getting married/having a relationship	5 (9.8)
	Other	15 (29.4)
	None of the above	5 (9.8)
**Positive impact on future plans**	Helped me decide on my vocation	2 (14.3)
	Helped my career	1 (7.1)
	Helped me know what I want out of life	8 (57.1)
	Helped me think about my financial future	9 (64.3)
	Other	5 (35.7)
	None of the above	1 (7.1)
**Social**		
**Impact on social health**	Not at all impactful	5 (6.3)
	Not very impactful	12 (15.0)
	Somewhat impactful	31 (38.8)
	Very impactful	20 (25.0)
	Extremely impactful	12 (15.0)
**Time off from work or studies**	Yes, from work	39 (48.8)
	Yes, from schooling/education	11 (13.8)
	Yes, from my leisure time	31 (38.8)
	No	22 (27.5)
	Does not apply	8 (10.0)
**Impact on work**	Caregiving makes it hard to travel for my job	11 (28.2)
	I've lost a job because of caregiving	5 (12.8)
	Caregiving has impacted my relationships with colleagues/boss	7 (17.9)
	I'm often late because of caregiving	13 (33.3)
	I can't focus because of caregiving responsibilities	18 (46.2)
	I'm unable to hold down a job because of caregiving responsibilities	5 (12.8)
	I can't dedicate as much time as I'd like to my job because of caregiving responsibilities	27 (69.2)
	Caregiving has taught me skills (including things like patience) that I use on the job	21 (53.8)
	I have to miss a lot of work days because of caregiving	10 (25.6)
	I make less money because of caregiving	14 (35.9)
	I've been overlooked for promotions because of caregiving	3 (7.7)
	Other	4 (10.3)
	None of the above	2 (5.1)
**Impact on education**	I can't focus because of caregiving responsibilities	3 (27.3)
	I'm often late because of caregiving responsibilities	1 (9.1)
	Caregiving responsibilities have left me struggling to catch up	4 (36.4)
	Caregiving responsibilities have impacted my relationships with fellow students/lecturers	0 (0.0)

	Caregiving responsibilities have affected my grades	3 (27.3)
	I can't dedicate as much time as I'd like to my schooling/education because of caregiving responsibilities	4 (36.4)
	I have to miss a lot of days because of caregiving responsibilities	1 (9.1)
	Hospital stay prevented me from going to school	2 (18.2)
	I had to withdraw from/drop out of school because of my caregiving responsibilities	3 (27.3)
	Other	0 (0.0)
	None of the above	2 (18.2)
**Impact on relation with HD patient**	It has brought us closer	25 (38.5)
	It has created resentment between us	14 (21.5)
	We argue more	14 (21.5)
	We are growing stronger together	15 (23.1)
	We know we will always have each other	30 (46.2)
	It has not impacted our relationship	3 (4.6)
	Other	10 (15.4)
	None of the above	0 (0.0)
**Impact on relation with others**	It led to my divorce	4 (5.0)
	It has prevented me from having a family	6 (7.5)
	It has prevented me from caring for other members of my family other than my (HD patient)	16 (20.0)
	I have lost some friends	28 (35.0)
	I am not able to socialize	21 (26.3)
	I am afraid to get into a long-term relationship	9 (11.3)
	It has made me realize how much people care	25 (31.3)
	It has made us closer	12 (15.0)
	I have made some new friends	21 (26.3)
	I often feel lonely/isolated	36 (45.0)
	I feel like I cannot discuss Huntington's Disease with other friends/family members	19 (23.8)
	Other	9 (11.3)
	None of the above	7 (8.8)

HD, Huntington's disease; CP, care partner.
